# A typical picture of Kayser-Fleisher ring in an 8-year-old child with Wilson's disease

**DOI:** 10.11604/pamj.2023.45.36.39057

**Published:** 2023-05-16

**Authors:** Mayuri Amol Deshpande, Amol Madhav Deshpande

**Affiliations:** 1 Department of Kayachikitsa, Mahatma Gandhi Ayurved College and Research Centre, Datta Meghe Institute of Higher Education and Research (Deemed to be University) Salod (H), Wardha, Maharashtra, India

**Keywords:** Kayser Fleisher ring, Indian childhood cirrhosis, Wilson’s disease

## Image in medicine

The mutations in the ATPP7B gene cause an autosomal recessive disorder known as Wilson's disease. It is also known as hepatolenticular degeneration and progressive lenticular degeneration. This disease, in the early stage, causes hepatic and neurogenic disorders. It may be present as hepatitis, cirrhosis, and hepatic decompensation. An episode of hepatitis may occur with or without jaundice which spontaneously regresses. Here is an image of Kayser-Fleisher ring which can be easily seen in a boy of age 8 years old. The boy was well oriented to space and time and didn´t show any symptoms except a brown-colored ring encircled cornea which is known as “Kayser-Fleisher ring” or “copper ring”. Before 4-6 months the boy was hospitalized for hepatitis.

**Figure 1:**
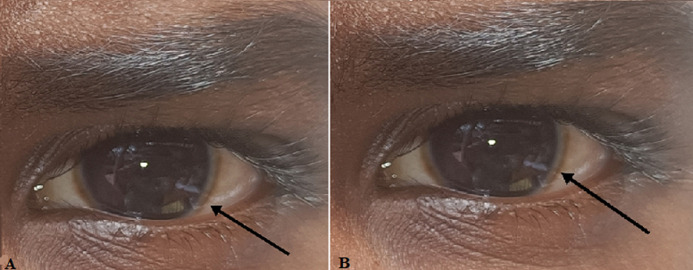
A) Kayser-Fleisher ring in right eye; B) Kayser-Fleisher ring in left eye

